# Supplementary material for: Leadership models in era of new technological challenges in construction projects
Source: PLoS One. 2022 Dec 15;17(12):e0278847. doi: 10.1371/journal.pone.0278847 (PMC9754181; doi:10.1371/journal.pone.0278847)
Supplement: S1 Data — (PDF) [file pone.0278847.s001.pdf]

No.	Gender	Age	Experience	Company_size	1-TF	2-TF	3-TF	1-TA	2-TA	3-TA	1-PA	2-PA	3-PA
1	F	27-33	less than 5 yrs	small	2	3	3	3	3	3	2	2	1
2	M	below 26	less than 5 yrs	large	2	3	3	4	2	3	2	2	2
3	M	below 26	less than 5 yrs	medium	2	3	4	4	3	3	3	1	1
4	F	below 26	less than 5 yrs	small	3	1	2	2	3	3	3	4	3
5	F	below 26	less than 5 yrs	large	2	2	1	3	1	2	1	0	1
6	M	34-40	10-15 yrs	medium	2	2	3	4	3	3	2	1	0
7	M	over 41	more than 15 yrs	large	4	3	4	3	0	0	4	4	4
8	F	27-33	less than 5 yrs	large	3	4	4	3	4	4	2	1	0
9	F	below 26	less than 5 yrs	medium	2	2	3	2	3	2	4	3	3
10	M	below 26	less than 5 yrs	small	2	2	2	3	3	3	2	1	1
11	F	below 26	less than 5 yrs	medium	2	3	3	3	2	2	2	1	2
12	F	27-33	less than 5 yrs	small	1	4	2	4	3	3	1	1	2
13	M	27-33	5-10 yrs	large	2	4	2	3	1	2	2	0	0
14	M	27-33	5-10 yrs	medium	3	4	4	4	4	4	1	0	0
15	M	34-40	5-10 yrs	medium	2	4	2	4	1	1	1	0	0
16	M	27-33	less than 5 yrs	medium	2	3	3	3	2	2	3	2	1
17	M	27-33	less than 5 yrs	large	2	3	3	1	3	3	3	1	1
18	M	over 41	more than 15 yrs	large	2	4	2	4	1	1	1	0	0
19	M	below 26	less than 5 yrs	small	4	3	3	3	3	3	1	1	1
20	M	34-40	5-10 yrs	small	3	3	3	2	2	2	3	2	1
21	M	27-33	5-10 yrs	medium	2	3	2	3	3	3	0	0	1
22	M	27-33	less than 5 yrs	large	2	3	4	3	3	3	2	1	1
23	M	34-40	10-15 yrs	medium	4	4	4	4	2	2	2	1	1
24	M	27-33	5-10 yrs	large	0	1	4	3	4	4	0	0	0
25	M	34-40	10-15 yrs	medium	3	3	3	4	2	3	2	0	1
26	M	over 41	more than 15 yrs	large	4	4	3	4	4	4	2	1	1
27	M	27-33	less than 5 yrs	large	2	2	4	3	3	3	1	2	1
28	M	34-40	10-15 yrs	medium	2	2	3	2	2	4	1	1	1
29	M	34-40	10-15 yrs	large	2	3	2	3	3	3	2	0	1
30	M	27-33	5-10 yrs	large	3	4	3	4	3	4	3	0	1
31	M	27-33	5-10 yrs	large	2	3	3	2	4	3	1	1	2
32	M	34-40	5-10 yrs	medium	2	3	3	3	3	2	1	1	1
33	M	below 26	less than 5 yrs	medium	1	4	4	3	3	3	3	1	1
34	M	34-40	5-10 yrs	large	2	2	3	3	3	2	3	1	1
35	M	27-33	less than 5 yrs	medium	2	2	2	2	2	2	2	2	2
36	M	below 26	less than 5 yrs	small	3	2	3	4	2	3	1	0	1
37	M	27-33	less than 5 yrs	large	1	4	2	2	3	3	2	1	2
38	F	over 41	more than 15 yrs	large	3	4	3	4	1	4	4	0	1
39	M	over 41	more than 15 yrs	large	3	4	4	3	3	3	2	1	1
40	M	27-33	5-10 yrs	large	2	3	2	2	3	3	2	2	2
41	M	27-33	5-10 yrs	large	2	3	2	3	2	2	1	1	1
