# Supplementary material for: Leadership models in era of new technological challenges in construction projects
Source: PLoS One. 2022 Dec 15;17(12):e0278847. doi: 10.1371/journal.pone.0278847 (PMC9754181; doi:10.1371/journal.pone.0278847)
Supplement: Attachment — Submitted filename: Reviewer 3.pdf [file pone.0278847.s002.pdf]

RESPONSE TO REVIEWER #3

Dear Reviewer,
As the authors of the original paper entitled "Leadership models in era of new technological challenges in construction projects", submitted to PlosOne, we would like to thank you for the feedback on our research.

COMMENTS:
The authors brought up an important question that how different kinds of leadership shape the digital transformation in construction industry. The beginning and ending part of this paper is well written. However, the methodology, questionnaire development and data analysis seemed to be separated parts from the rest of the paper. For example, how simple analysis of distributions and averages will illustrate the influence of leadership types? Is there a significance analysis to argue for statistically significant results? Will the size of companies has an impact on leadership types and technology developments? Hope the authors could explain further in the future.

REPLY:
Your comments motivated us to fully rebuild our manuscript. We added several crucial references to eliminate the gaps.
We hope you are satisfied with the updated version.

- The abstract and keywords were changed.
- The introduction was modified to underline what we concentrate on.
- The second chapter was changed.
- The third chapter was modified according to your suggestions.
- We hope the modified Conclusions are within the research findings.

We hope our explanations make the manuscript more consistent.

- Authors
