# Supplementary material for: Leadership models in era of new technological challenges in construction projects
Source: PLoS One. 2022 Dec 15;17(12):e0278847. doi: 10.1371/journal.pone.0278847 (PMC9754181; doi:10.1371/journal.pone.0278847)
Supplement: Attachment — Submitted filename: Response to Reviewers - 2.docx [file pone.0278847.s003.docx]

Dear Reviewers,

As the authors of the original paper entitled “Leadership models in era of new technological challenges in construction projects”, submitted to PlosOne, we would like to thank you for the feedback on our research. We feel that the quality of the article has improved thanks to your valuable review contribution. Indeed, there are still some issues that we would like to address in this round of revision.

**RESPONSE TO REVIEWER #2**

COMMENTS:

The authors have addressed most of the reviewers' concerns. However, they should take the following questions into consideration:

1. They need to explain how the 9 questions reflect the three kinds of leadership models. Is there existing research that validated the scale? Are the three questions enough to describe the leadership models?

2. There is lack of explanation of how the questionnaire results are related to the leadership model proposed in Section 5.1.

REPLY:

The multiple leadership questionnaire (MLQ) scale developed by Bass and Avolio and used by one of the authors in his paper [Keleş et al, 2021] was used as the basis for determining the questionnaire questions of this study [Bass and Avolio, 1995]. The questionnaire defines the 3 leadership types discussed in this study and consists of 45 statements on a 5-point Likert scale. In the research, 9 of 45 questions, which were the most decisive, were selected and asked the participants during a session of the questionnaire. In this respect, it is clear that the answers to these questions can be used in determining the leadership types aimed at the study. This information has been added to the "Materials and Methods" section (lines 295-301).

As a result of the survey, it is seen that "transactional" and "transformational" leadership types dominate over “passive/avoidant” patterns, however, what was revealed, there is no single best ‘one-fits-all’ leadership approach that could be used within a random construction project organization. On the other hand, this is in line with the evolutionary leadership model [Hersey and Blanchard, 1969], being an effective knowledge diffusion tool and an enabler for improving the maturity [Serna, 2015] of technological issues in a construction company, what was proposed in this study. In this respect, it is thought that the dissemination of the study results in the construction sector will contribute to the knowledge at a global scale. Such an explanation was added in lines 536-543.

Thank you for your valuable contribution. We believe our manuscript has been enriched even more.

- Authors
